# Supplementary material for: Stacking Model‐Based Classifiers for Dealing With Multiple Sets of Noisy Labels
Source: Biom J. 2025 Mar 12;67(2):e70042. doi: 10.1002/bimj.70042 (PMC11898607; doi:10.1002/bimj.70042)
Supplement: Supplementary file 1 — Supporting Information [file BIMJ-67-e70042-s001.zip › density-based-ensemble-model-main/competitors_real_data/netlab-master/netlab-master/help/consist.htm]

Netlab Reference Manual consist


# consist

## Purpose

Check that arguments are consistent.

## Synopsis

```
errstring = consist(net, type, inputs, outputs)
errstring = consist(net, type, inputs)
errstring = consist(net, type)
```

## Description

`errstring = consist(net, type, inputs)` takes a network
data structure `net` together with a string `type` containing
the correct network type, a matrix `inputs` of input vectors and checks
that the data structure is consistent with the other arguments. An empty
string is returned if there is no error, otherwise the string contains the
relevant error message. If the `type` string is empty, then any
type of network is allowed.

`errstring = consist(net, type)` takes a network data structure
`net` together with a string `type` containing the correct
network type, and checks that the two types match.

`errstring = consist(net, type, inputs, outputs)` also checks that the
network has the correct number of outputs, and that the number of patterns
in the `inputs` and `outputs` is the same. The fields in `net`
that are used are

```
  type
  nin
  nout
```

## Example

`mlpfwd`, the function that propagates values forward through an MLP
network, has the following check at the head of the file:

```
errstring = consist(net, 'mlp', x, t);
if ~isempty(errstring)
  error(errstring)
end
```

## See Also

`mlpfwd`

---

**Pages:**
Index


---

Copyright (c) Ian T Nabney (1996-9)
